# Improving stereotactic radiotherapy (SRT) planning process for brain metastases by Cyberknife system: reducing dose distribution in healthy tissues

**DOI:** 10.7150/jca.41102

**Published:** 2020-04-13

**Authors:** Yu Xuyao, Yuan Zhiyong, Wang Yuwen, Yu Hui, Song Yongchun, Dong Yang, Zhao LuJun, Wang Ping

**Affiliations:** 1 Department of Radiotherapy, Tianjin Tumour Hospital, Tianjin, China; 2 Biomedical Engineering, Tianjin University, Tianjin, China

**Keywords:** CyberKnife, stereotactic radiotherapy, brain metastases, treatment plan, prescription dose line

## Abstract

**Purpose:** To pursue high precision dose in lesions and steeper dose fall-off in healthy tissues of brain metastases stereotactic radiotherapy (SRT), this study investigated an opitimized planning by comparison different prescription dose line in the treatment of brain metastases using Cyberknife (CK) Robotic Radiosurgery System.

**Methods:** 77 patients (92 lesions) brain metastases patients CK SRT plans were replanned with 50%-80% (5% internal) prescription dose line to cover more than 95% of the planned target volume (PTV), under the same collimator by Multiplan System. Under the precondition of guaranteeing plans all meet the clinical requirements, the plan evaluation paraments (conformal index (*CI*) and homogeneity index (*HI*)), plan treatment time parameters (the total number of beams and monitor units (MU)) and dose distribution of organs at risk (OAR) and healthy brain tissues adjacent to the PTV were analyzed respectively.

**Resluts:** Compared with 70% plans, 65% plans had: 1) average dose (*D_mean_*) and maximum dose (*D_max_*) of healthy brain tissue outside of the PTV reduced 11.83% and 5.97% markedly; 2) *D_mean_* and *D_max_* of brainstem decreased 11.43% and 2.86%; 3) the volumes of whole brain minus the tumors received a single dose equivalence of 12 Gy/14 Gy (V12Gy/V14Gy) had marked decline. The dose fall-off was considerably faster in the 60%-65% plans around the PTV and the maximum dose of healthy tissue was prominently lower. While the difference in *CI* and *HI* between different plans was not obvious, the plan treatment time was a little higher in 60%-65% plans than 70%-80% plans.

**Conclusions:** Choosing a relatively lower isodose as the prescription dose line for brain metastases CK SRT planing could improve the dosimetry index of target and immensely reduce high dose in healthy brain tissue and OAR.

## Introduction

With improvements in control of systemic disease, better radiologic detection, and prolonged survival, the brain metastases in patients with cancer are more frequent than primary brain tumors 1. Brain metastasis occurs in approximately 20-40% of patients with cancer, and that has become an important factor affecting the survival of patients 2. Numerous studies have shown stereotactic radiotherapy (SRT) has better local control (LC) of the treated brain metastases than surgery, and solves the shortcomings of whole brain radiotherapy (WBRT) including low dose distribution in the tumor area and high side effects. SRT has been known as the best available treatment for cancer patients with one to three brain metastases 3.

CyberKnife (CK) SRT system uses real-time X-ray exposure to locate the patients' head, and the linear accelerator is controlled by the six-dimensional robotic arm for precise positioning radiotherapy according to the results of the tracking algorithm. That can effectively improve SRT treatment accuracy and reduce the damage to healthy tissues 4,5,6. Therefore, CK SRT has been widely used for 1-3 brain metastases patients clinical treatment.

Park S H et al. 7 used Gamma knife radiosurgery for multiple brain metastases with mean prescription isodose at the tumor margin was 50% (range: 40%-60%). Sio T T et al. 8 adopted the 80% isodose line for the prescribed dose in the CK SRS plans for patients with brain metastases. Takashi U et al. 9 selected 59%-79% prescribed isodose line for head tumors CK planning. Zindler J D et al. 10 studied stereotactic radiosurgery for 4-10 brain metastases and considered dose inhomogeneity, with a maximum allowed dose within the planning target volume (PTV) of 140% prescribed dose (70% prescription isodose).

Isodose line from 50% to 80% isodose is often chosen as the prescribed dose line for brain metastases CK SRT planning in clinical practice. There is very little related researches about the effect of prescription dose line on dosimetry distribution in healthy brain tissues during CK SRT planning in detail, and which is not any uniform standard.

Several studies show that higher total irradiation dose, fraction dose and irradiated volume of healthy brain tissue adjacent to lesions are associated with more clinically meaningful cases of cerebral radiation necrosis (CRN) 11. During CK SRT planning, choosing different isodose line of global maximum as the prescribed dose curve will not only vary the maximum dose values in the tumor area, and cause different dose distribution of organs at risk (OAR) and healthy brain tissue. However, previous studies have shown that CRN is related to dose distribution in healthy brain tissue around the PTV and V12/V14 (surrounding brain volume circumscribed with a single dose equivalence of 12/14 Gy.

Therefore, in this paper, we conducted a retrospective study to analyzed dosimetry distribution of healthy tissues in CK SRT plans for brain metastases with difference prescription dose line, 50%-80% (5% internal) isodose, and the purpose of it was to identify the optimal prescription isodose line for improving CK treatment: 1) to deliver large dose to the tumor, 2) achieve minimizes the amount of radiation delivered the healthy tissues.

## Material and Methods

### Patient characteristics

From January 2016 to June 2019, 77 patients were treated with image guided SRT using CK system (CyberKnife Ⅲ, Accuray Inc., Sunnyvale, CA) for brain metastasis) at Tianjin Medical University Cancer Institute and Hospital. All patients participating in this study had not metastases to other oragans and not undergone pre- CK SRS metastasectomy or pre- or concurrent-to- CK SRS WBRT. Twenty-three patients (29.9%) had a KPS of<70 and all patients were in RTOG-RPA class 2 or 3. This report analyzed a head-to-head, quantitative comparison of dosimetry profiles between the different treatment plans for 92 brain metastases lesions (15 patients with two brain metastatic lesions (included 30 lesions)) by Multiplan system (Accuray, Sunnyvale, CA, USA) software program, which determines the lesions volume based on computed tomography (CT) and enhanced T1-weighted magnetic resonance imaging (MRI) fusion. Table 1 shows the patient characteristics.

### Target delineation and CK SRT treatment planning

Overall skull CT scan for the patients was performed by Philips Brilliance Big Bore CT (16 rows), with the thickness of scan layer of 1.5 mm. T1-weighted magnetic resonance imaging scan with Siemens 1.5 was registered to CT. CT and MRI images were used for the delineation of the gross tumor volume (GTV) and the critical organ structures (also called OAR) including brainstem, eyes, lens, optic nerve, optic chiasm, and pituitary gland. The targeting error of brain CK SRT under skull tracking is 0.956 mm, a 1.6-mm margin was added to the GTV to create the plan tumor volume (PTV), expects the patients with brainstem metastasis 12.

Five dose-limiting shells (2 mm, 3 mm, 5 mm, 7 mm, 9 mm) away from the each PTVs were created to optimize the dose distribution to healthy brain tissues. Based on the delineation results and requirements, the planner conducted the design and optimization of the CK SRT plans by adopting the reverse optimization and nonisocentric algorithm through the MultiPlan system. A high-resolution calculation step was performed in the evaluation step to finalize the CK SRT plans. For each patient, seven different CK SRT plans were designed by using different prescription isodose ranged from 50% to 80% (including Plan_50%, Plan_55%, Plan_60%, Plan_65%, Plan_70%, Plan_75%, Plan_80%). The prescription isodose must cover more than 95% of the PTV volume in all plans. All of the plans for the same patient followed the same dose limit conditions of OAR, without the iris or MLC system, in order to ensure the consistency of beam data in plans. During CK SRT Plannging for 15 patients with two brain metastatic lesions (included 30 lesions), the prescription dose line of each lesions plans should be consistent. And high-resolution calculation step was performed in the evaluation step to finalize CK SRT plans.

### Evaluation of CK SRT plans

Firstly, the minimum dose (*D_min_*), maximum dose (*D_max_*) and mean dose (*D_mean_*) were evaluated and compared in the PTV, OAR and healthy brain tissue around the PTV (PTV+2 meant 2-mm-thick healthy brain tissue adjacent to the PTV, PTV+6 meant 4-mm-thick healthy brain tissue adjacent to the PTV+2). Secondly, the volume of whole brain tissue minus the PTV received 12 Gy/14 Gy (V12Gy/ V14Gy) in a single fraction was calculated by the LQ model. Finally, the plan evaluation paraments (conformal index (*CI*) and homogeneity index (*HI*)) and the plan treatment time parameters (the total number of beams and monitor units (*MU*)) were compared.

*CI* was commonly used to evaluate CK SRT plans 13 and calculated as follows:

*CI*=*PIV*/*TIV* (1)

Where *PIV* was the volume included by prescription isodose, and *TIV* was the tumour volume covered by prescription isodose volume. This definition of *CI* is different than the radiation therapy oncology group (RTOG) definition, which is *PIV* divided by total tumor volume 14. The closer the value of *CI* is 1, the better the plan.

## Results

**Figure 1:**
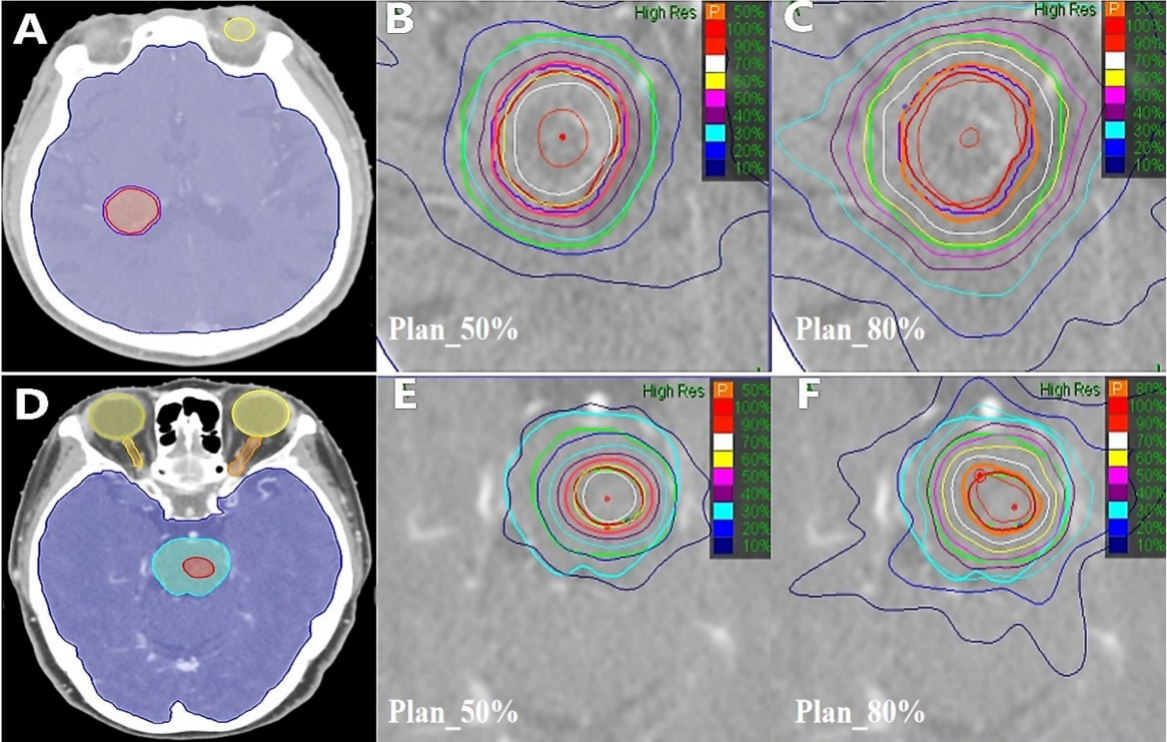
** Different CK SRT plans for brain metastases patients.** The representative patient had axial images taken, **(A)** and **(D)** were Patient 1 with metastases in the right lateral ventricle and Patient 2 with brainstem metastases. The red and purple lines area indicate the GTV and the PTV, respectively. Blue, orange and yellow lines represent Brainstem, Optic Nerves, and Eyes, respectively. **(B)** and **(E)** represent 50% prescription isodose covered more than 95% of the Patient 1 PTV and Patient 2 GTV (Plan_50%). **(C)** and **(F)** represent 80% prescription isodose covered more than 95% of the Patient 1 PTV or Patient 2 GTV (Plan_80%).Green line zones were covered 6-mm thick healthy brain tissue adjacent to the PTV or the GTV.

The results of different CK SRT plans using 50% and 80% prescription isodose for two patients (Patient 1 with metastases in the right lateral ventricle, Patient 2 with brainstem metastases) were shown in Figure 1.

These results illustrated that 1) the radiation around PTV was more divergent with the value of the prescription dose line increased from 50% to 80%. For example, the 30% isodose (blue line) was included in the PTV+6 area in Plan_50% (as shown in Figure.1(B) and (E)), but not in Plan_80% (as shown in Figure.1(C) and(F)). 2) OAR (notably, the brainstem) and healthy brain tissue were characterized as less irradiated areas and had a closed isodose in Plan_50%. These illustrated that using lower prescription isodose could significantly reduce dose distribution in plans.

**Figure 2:**
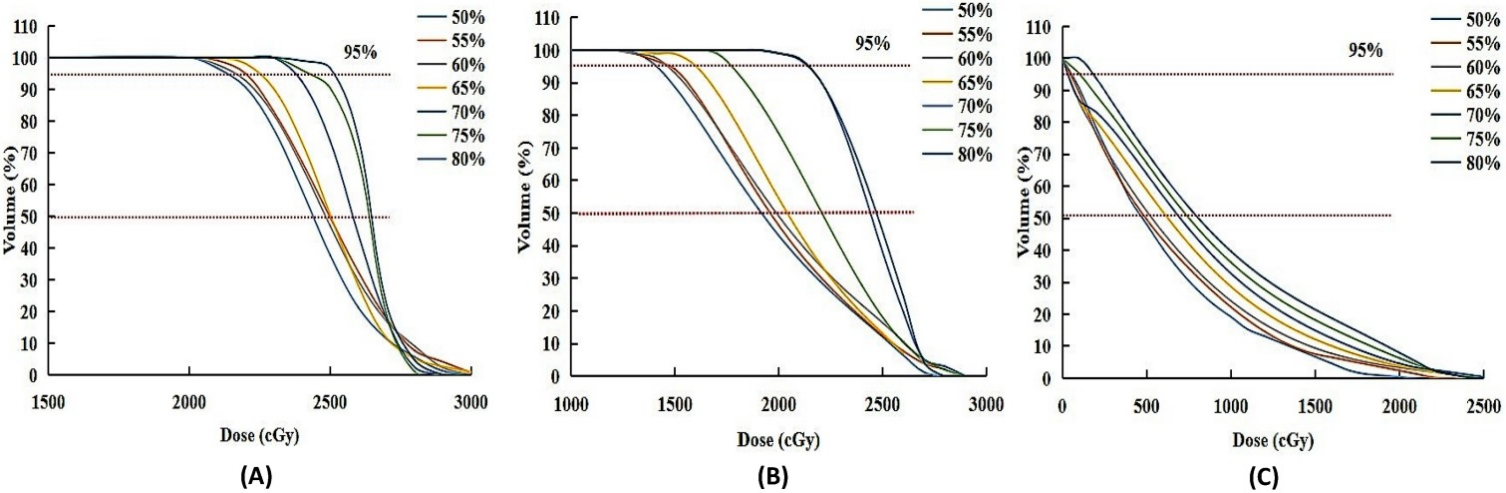
** PTV+2, PTV+6 and brainstem dose volume histogram (DVH) of patient 2 with brainstem metastases. (A)** and **(B)** were dosimetry distribution of 2-mm-thick and 6-mm-thick healthy brain tissue adjacent to the PTV. **(C)** was the dosimetry distribution of Brainstem.

In order to quantify the difference in dose distribution, PTV+2, PTV+6 and brainstem dose volume histogram (DVH) of patient 2 were compared in Figure 2. Although Plan_80% had the maximum slope of DVH, the dose covering more than 50% and 95% volume of the PTV+2 and PTV+6 area was the biggest in it, especially. *D_mean_* was kept in a lower range in Plan_50% to Plan_65%. The same result was found in the brainstem region, especially the dose covering more than 50% volumes of it. These demonstrated that more healthy brain tissue and OAR adjacent tumor tissue received higher dose in CK SRT plans with higher prescription dose line.

### Statistical analysis of dosimetric distribution

The dosimetric distribution in CK SRT plans for 92 intracranial tumours were shown in Table 2. The value of *D_min_*, *D_max_* and *D_mean_* were expressed as percent of the global maximum dose in plans. The *D_min_* and *D_mean_* of the PTV were slightly higher with increasing value of prescription isodose from 50% to 80%. In Plan_65%, *D_mean_* and *D_max_*of the PTV+6 were significantly reduced by 11.83% and 5.97%, compared with Plan_70%. These results further confirmed that the dosimetric distribution exhibited better convergence and healthy brain tissues around the PTV received a lower radiation dose in Plan_65%.

The dosimetry distribution of OAR was shown in Table 3. The Dmax and Dmean of the optic chiasm and optic nerve were lower in plans with lower isodose as the prescription dose curve. Compared with Plan_70%, the Dmean and Dmax dose of the brainstem were reduced 11.43% and 2.86% respectively in Plan_60% for patients with brainstem metastasis. The brain metastasis selected in this study was located far from the eyes, and the eyes (especially lens) had the highest protection priority during the CK SRT plan designing. Thus, the dosimetry distribution of the lens was the lowest and not significantly different between plans.

The PTV Coverage, *HI*, *MU* and *CI* of different CK SRT plans for 77 patients were statistically analyzed, and the results were shown in Table 4. PTV Coverage of different CK SRT plans was similar. It indicated that the PTV dose cloud meet the requirement of the clinical dose prescribed in each plan.

The *CI* and* HI* of Plan_50% and Plan_55% were significantly different from other plans. That was because it required a bigger collimator to improve dosimetry distribution of the PTV under the same other condition. While it could also lead to uneven dosimetry distribution in the PTV area, Plan_50% had very hot spots which was not only a spot, but could be a discrete tumor volume. And the machine hop of Plan_50% and Plan_55% were higher than other plans. While the difference of total beam counts between plans was not significant. Therefore, Plan_50% and Plan_55% had the longer treatment time.

**Figure 3:**
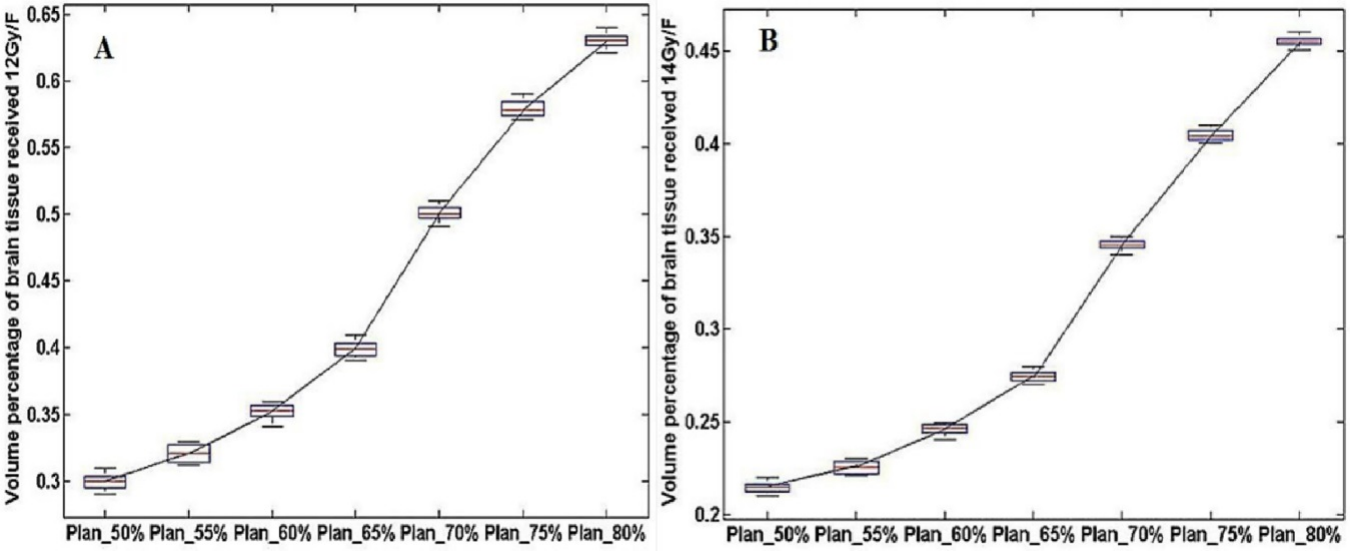
** The dosimetry distribution of the whole brain tissue in different plans for the same patients. (A)** and **(B)** were the volume of healthy brain tissue received 12 Gy/14 Gy (V12Gy/V14Gy) in a single fraction.

The statistical results of V12Gy/V14Gy were shown in Figure 3. It could be observed that V12Gy/ V14Gy value had a marked decline in Plan_65%. This finding provides more evidence for the theory that CK SRT plans with 65% isodose as prescribed dose curve could protect healthy brain tissue better while satisfying the need for clinical treatment.

SRT is used extensively to treat the brain metastases patients who are not clinically suitable for surgery or experience postoperative recurrence 15. Compared with traditional radiotherapy, SBRT exhibits higher local control rates, effectively reduces intracranial exposure doses, minimizes nerve function injury in patients, and better protects normal brain tissue. CK SRT can achieve high treatment accuracy given its real-time skull matching and tracking function 16,17. However, due to the single high exposure dose caused by the hypofractionated radiotherapy, it is very easy to cause radiation-induced brain injury.

Three-fraction (tumors>10 cm^3^) and five-fraction (large tumors>30 cm^3^) CK radiotherapy for brain metastases recommended requiring tight control a single dose equivalent of 14 Gy (V14 Gy) to avoid radiation necrosis in patients with metastases 18. More strict conclusions, the irradiated volume of healthy brain tissue with a single dose equivalent of more than 12 Gy (V12 Gy) was a predictor of brain radio necrosis. The 12 Gy volume of the brainstem is recommended to be decreased to as low as possible in single fraction radiosurgery to reduce the occurrence of any adverse radiation imaging effects on the brainstem and to avoid new neurological deficits 19. During the CK SRT planning, using different prescription isodose would affect dosimetry distribution in the healthy brain tissue of brain metastases patients. At present, the isodose between 50% and 80% will be selected as the prescription dose and there is no uniform standard for it 20,21. In this study, the adoption of the method in which using lower prescription isodose during the development of CK SRT plans could effectively reduce the exposure dose of healthy brain tissue around the PTV and the OAR. However, with utilization of lower normalized isodose as the prescription dose curve, a higher volumetric global maximum was observed. This approach may decrease uniformity of dose distribution and cause necrosis in the PTV volume.

## Conclusion

In this paper, different CK SRT plans for the same patients with brain metastases were designed with different prescription dose line. Although, selecting lower isodose as prescription dose line can increase high dose in tumors and reduce dose delivered to the healthy brain tissues and OAR. But the Plan_50% and Plan_55% often needed bigger collimator and had longer treatment time, and higher* CI* and *HI*. While Plan_50% and Plan_55% had very hot spots which was not only a spot, but could be a discrete tumor volume. Therefore, it is best to choose 60-65% isodose line as the prescription dose line covered more than 95% volume of the PTV during CK SRT planning. That could satisfy the need of clinical treatment, while reducing the dosimetry distribution of healthy brain tissue and OAR. These guidelines offer a good protective effect for patients and yield a certain clinical reference value. However, this study is still based on the analysis of the CK SRT planning. Further follow-up of patients who undergo treatment, and statistical analysis of radiation-induced brain necrosis in different clinical design plans should be conducted to provide more powerful evidence for this study and improve the therapeutic effect of patients with brain metastases by CK SRT treatment.

## Figures and Tables

**Table 1:** Clinical characteristics of 77 patients.

**Median age (years)(range)**	53(27-81)
Age≥60	28
Age<60	49
**Sex**	
Male	32
Female	45
**Primary cancer**	
Liver	15
Breast	20
Lung	45
**Median KPS score**	72(51-100)
KPS≥70	54
KPS <70	23
**Multiple vs. Single**	
Multiple metastases	15
Single metastasis	62
**Location of tumor**	
Cerebral hemisphere	47
Cerebellum	35
Brainstem	10
**Tumor volume (cm^3^) (range)**	4.6(0.05-9.89)
5.0-10.0	17
1.0-5.0	64
<1.0	11
**Prescription dose(Gy)(range)**	12-32(24)
**Fraction(range)**	1-4(3)

**Table 2:** The dose distribution of healthy brain tissue around the PTV (using percent of the global maximum dose).

	PTV			PTV+2			PTV+6	
	*D_mean_*	* D_min_*		*D_max_*	*D_mean_*		*D_max_*	*D_mean_*
Plan_50%	63.62%±3.80%	53.51%±1.46%		57.28%±3.20%	47.57%±2.56%		53.40%±3.11%	35.86%±2.03%
Plan_55%	65.13%±2.71%	54.61%±1.96%		58.02%±4.01%	48.50%±2.17%		56.12%±3.49%	36.24%±1.96%
Plan_60%	67.34%±2.92%	55.08%±1.73%		60.25%±3.14%	50.09%±2.87%		58.84%±2.99%	39.8%±3.23%
Plan_65%	68.26%±3.16%	56.12%±2.04%		65.45%±3.28%	51.27%±3.58%		63.35%±3.50%	39.96%±3.67%
Plan_70%	70.82%±2.75%	62.39%±1.80%		69.63%±3.77%	54.47%±4.01%		67.37%±3.37%	45.32%±2.15%
Plan_75%	71.94%±3.03%	63.61%±1.54%		70.82%±3.65%	57.12%±3.44%		69.34%±2.86%	53.42%±4.21%
Plan_80%	76.56%±3.17%	69.10%±1.74%		72.49%±3.09%	62.06%±3.97%		70.88%±3.24%	57.35%±2.65%

*PTV+2 meant 2-mm-thick healthy brain tissue adjacent to the PTV, PTV+6 meant 4-mm-thick healthy brain tissue adjacent to the PTV+2

**Table 3:** The dosimetry distribution of OAR (using percent of the global maximum dose).

	Brainstem			Optic Nerves			Optic Chiasm	
	*D_max_*	*D_mean_*		*D_max_*	*D_mean_*		*D_max_*	*D_mean_*
Plan_50%	63.62%±3.17%	20.75%±1.55%		14.84%±4.17%	7.51%±3.76%		18.84%±4.07%	5.39%±1.17%
Plan_55%	65.13%±2.44%	21.20%±1.34%		15.05%±2.98%	7.59%±2.91%		19.25%±4.30%	5.44%±1.49%
Plan_60%	67.34%±3.09%	22.13%±1.46%		16.12%±3.70%	7.62%±2.95%		20.43%±4.12%	5.80%±1.96%
Plan_65%	68.26%±2.91%	22.40%±1.19%		16.45%±3.24%	8.03%±3.12%		22.17%±3.37%	6.11%±2.31%
Plan_70%	70.82%±2.84%	25.29%±2.07%		19.05%±2.78%	8.23%±3.51%		23.75%±3.37%	7.28%±2.15%
Plan_75%	71.94%±3.15%	29.02%±2.13%		20.46%±3.14%	9.25%±3.18%		24.73%±2.16%	7.42%±1.98%
Plan_80%	76.56%±2.97%	31.25%±1.83%		22.49%±3.09%	10.06%±3.62%		25.88%±3.24%	7.51%±2.14%

*Brainstem dose statistics were from 10 patients(10 lesions) with brainstem metastasis. Optic nerves were superposition of both sides.

**Table 4:** The statistical result of index for different plans

	PTV* Coverage*	*Conformal index* (*CI*)	*Homogeneity index* (*HI*)	*Total Beam counts*	*Machine hop(MU)*
Plan_50%	96.02%±0.81%	1.20±0.10	1.41±0.37	108±11	7276±256
Plan_55%	95.83%±0.52%	1.20±0.09	1.40±0.39	102±8	7154±217
Plan_60%	96.00%±0.74%	1.15±0.07	1.36±0.21	105±14	6990±187
Plan_65%	95.98%±0.36%	1.16±0.09	1.36±0.30	110±12	6951±135
Plan_70%	96.12%±0.29%	1.14±0.08	1.34±0.28	106±9	6869±211
Plan_75%	95.69%±0.38%	1.15±0.09	1.35±0.33	113±15	6834±158
Plan_80%	95.84%±0.37%	1.16±0.07	1.33±0.32	109±12	6738±167

^*^The value of machine hop were in single fraction radiosurgery.
